# Supplementary material for: MetaQTL: a package of new computational methods for the meta-analysis of QTL mapping experiments
Source: BMC Bioinformatics. 2007 Feb 8;8:49. doi: 10.1186/1471-2105-8-49 (PMC1808479; doi:10.1186/1471-2105-8-49)
Supplement: Additional File 7 — MetaQTL Package : jar file and tutorial. This Zip archive contains both the MetaQTL JAR file and the files of the tutorial. [file 1471-2105-8-49-S7.zip › org.inra.metaqtl/doc/org/metaqtl/adapter/QtlPartitionAdapter.html]

QtlPartitionAdapter


|  |  |  |
| --- | --- | --- |
| **Overview** \| **Package** \| **Class** \| **Use** \| **Tree** \| **Deprecated** \| **Index** \| **Help** | |  |
| **PREV CLASS**   NEXT CLASS | **FRAMES**    **NO FRAMES**     **All Classes** |  |
| SUMMARY: NESTED \| FIELD \| CONSTR \| METHOD | DETAIL: FIELD \| CONSTR \| METHOD |  |


---


## org.metaqtl.adapter Class QtlPartitionAdapter

```
java.lang.Object
  org.metaqtl.adapter.QtlPartitionAdapter
```

---

``` public class QtlPartitionAdapter extends java.lang.Object ```

This class defines some methods to adapt complient
objects into a `QtlPartition` object.

---

**Constructor Summary**

`QtlPartitionAdapter()`


| **Method Summary** | |
| --- | --- |
| `static QtlPartition` | `adapt(MetaQtlAnalysis analysis, java.lang.String chrom, java.lang.String trait, int k)`             Adapt the `MetaQtlAnalysis` `analysis` for a given value of the number of clusters `k` and a given trait name `code`. |
| `static QtlPartition` | `adapt(MetaQtlData qtlData)`             Adapt the given `MetaQtlData` into a `QtlPartition` and returns it. |
| `static QtlPartition` | `adapt(MetaQtlResult result, Qtl[] qtls, int k)` |

**Methods inherited from class java.lang.Object**

`clone, equals, finalize, getClass, hashCode, notify, notifyAll, toString, wait, wait, wait`

**Constructor Detail**

### QtlPartitionAdapter

```
public QtlPartitionAdapter()
```


**Method Detail**

### adapt

```
public static QtlPartition adapt(MetaQtlData qtlData)
```

:   Adapt the given `MetaQtlData` into a
    `QtlPartition` and returns it.

    :   **Parameters:**: `qtlData` - the `MetaQtlData` instance to adapt. **Returns:**: a new instance of `QtlPartition`.

---


### adapt

```
public static QtlPartition adapt(MetaQtlAnalysis analysis,
                                 java.lang.String chrom,
                                 java.lang.String trait,
                                 int k)
```

:   Adapt the `MetaQtlAnalysis` `analysis` for
    a given value of the number of clusters `k` and a
    given trait name `code`.
    into a new instance of `QtlPartition`.

    :   **Parameters:**: `result` - a meta-analysis result.: `k` - the number of clusters. **Returns:**: a new instance of `QtlPartition` with as many partitions as the value of `k`.

---


### adapt

```
public static QtlPartition adapt(MetaQtlResult result,
                                 Qtl[] qtls,
                                 int k)
```


---


|  |  |  |
| --- | --- | --- |
| **Overview** \| **Package** \| **Class** \| **Use** \| **Tree** \| **Deprecated** \| **Index** \| **Help** | |  |
| **PREV CLASS**   NEXT CLASS | **FRAMES**    **NO FRAMES**     **All Classes** |  |
| SUMMARY: NESTED \| FIELD \| CONSTR \| METHOD | DETAIL: FIELD \| CONSTR \| METHOD |  |


---
